# Current status and progress of PD-L1 detection: guiding immunotherapy for non-small cell lung cancer

**DOI:** 10.1007/s10238-024-01404-1

**Published:** 2024-07-18

**Authors:** Chang Qi, Yalun Li, Hao Zeng, Qi Wei, Sihan Tan, Yuanyuan Zhang, Weimin Li, Panwen Tian

**Affiliations:** 1 Department of Pulmonary and Critical Care Medicine, State Key Laboratory of Respiratory Health and Multimorbidity, Institute of Respiratory Health and Multimorbidity, Institute of Respiratory Health, Frontiers Science Center for Disease-related Molecular Network, Precision Medicine Center/Precision Medicine Key Laboratory of Sichuan Province, West China Hospital, Sichuan University, Chengdu, Sichuan China; 2 Lung Cancer Center/Lung Cancer Institute, West China Hospital, Sichuan University, Chengdu, Sichuan China

**Keywords:** Non-small cell lung cancer, Programmed cell death 1 ligand 1, Immunohistochemical, Liquid biopsy, Exosome

## Abstract

Non-small cell lung cancer (NSCLC) is the leading cause of cancer-related deaths and represents a substantial disease burden worldwide. Immune checkpoint inhibitors combined with chemotherapy are the standard first-line therapy for advanced NSCLC without driver mutations. Programmed death-ligand 1 (PD-L1) is currently the only approved immunotherapy marker. PD-L1 detection methods are diverse and have developed rapidly in recent years, such as improved immunohistochemical detection methods, the application of liquid biopsy in PD-L1 detection, genetic testing, radionuclide imaging, and the use of machine learning methods to construct PD-L1 prediction models. This review focuses on the detection methods and challenges of PD-L1 from different sources, and discusses the influencing factors of PD-L1 detection and the value of combined biomarkers. Provide support for clinical screening of immunotherapy-advantage groups and formulation of personalized treatment decisions.

## Introduction

Lung cancer is one of the most common and one of the most deadly malignancies worldwide and is often diagnosed at an advanced stage due to its insidious onset, which leads to a poor prognosis [1, 2]. In recent years, with advancements in antitumor treatment modalities and the emergence of new drugs, immunotherapy has become the standard first-line treatment for patients with advanced non-small cell lung cancer (NSCLC) harboring negative driver mutations and can significantly improve long-term survival rates [3]. Programmed death-ligand 1 (PD-L1) is currently the only prognostic biomarker recommended by the National Comprehensive Cancer Network for immunotherapy (NCCN), used to formulate treatment strategies for metastatic NSCLC [4].

However, the clinical implementation of PD-L1 evaluation presents several challenges that need to be addressed to optimize its diagnostic utility and therapeutic potential. One primary challenge is the intratumoral heterogeneity (ITH). The ITH complicates assessment and necessitates multiple sampling points to obtain a representative analysis. Additionally, PD-L1 expression in tumors is a systemic and dynamic process, which can be influenced by various factors such as treatment modalities, tumor microenvironment, and inflammatory responses. Repeated evaluations are necessary to accurately capture the PD-L1 status. However, the invasive nature of the sampling process and the limitations of local sampling currently hinder clinical PD-L1 testing from capturing the temporal and spatial heterogeneity of tumors. Moreover, the detection of PD-L1 lacks standardized detection protocols. The antibodies used for detection are susceptible to variations in detection methods, platforms, and post-translational modifications (PTM) of the PD-L1 protein. And there is ongoing controversy regarding the definition of clinically relevant cutoff values for PD-L1 positivity. These discrepancies impede the establishment of universally accepted guidelines and impact treatment decisions.

**Fig. 1:**
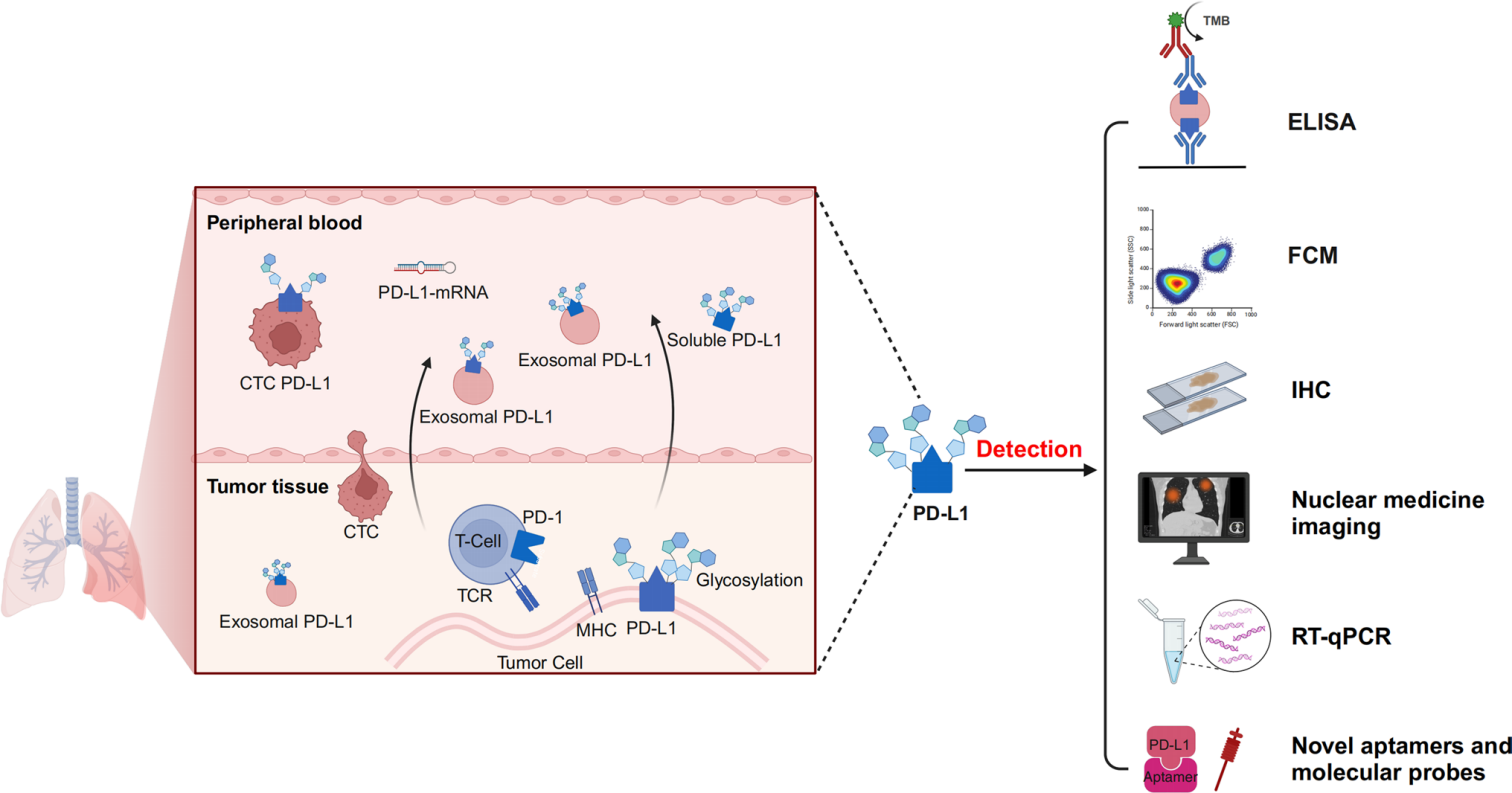
Detection methods and protein expression of PD-L1 in NSCLC

Therefore, it is necessary to select appropriate detection methods and markers before immunotherapy. As researchers’ understanding of PD-L1 protein expression in tumor patients has deepened, the methods for detecting PD-L1 have also evolved. More specific and higher-affinity antibodies and improved immunohistochemistry (IHC) technology eliminate interfering factors and increase the stability of detection. The development of liquid biopsy and nuclear imaging technology has made comprehensive and multidimensional PD-L1 detection a reality. In this review, we provide an overview of various methods for detecting PD-L1 and their clinical application in NSCLC patients based on the different sources of PD-L1 in the body (Fig. 1).

## Clinical significance of PD-L1

In the clinical setting, PD-L1 protein expression has become a useful biomarker for decision-making in lung cancer immunotherapy treatment. The Keynote-001 study evaluated the safety and effectiveness of Pembrolizumab in the treatment of advanced NSCLC patients and confirmed that the level of PD-L1 expression was related to clinical benefit [5]. CheckMate 057 is a randomized, open-label Phase III clinical trial. Patients with non-squamous NSCLC who had received platinum-based doublet chemotherapy were treated with Nivolumab and docetaxel, respectively. Patients were divided into sub-groups based on PD-L1 expression levels (≥ 1%, > 5%, > 10%). The results showed that Nivolumab was more effective in patients with positive PD-L1 expression, and in all sub-groups, it demonstrated better efficacy than docetaxel [6]. The OAK trial found that patients receiving atezolizumab with high PD-L1 expression (≥ 1% PD-L1 on tumor cells or tumor-infiltrating immune cells (TIL)) in the tumor microenvironment (TME) had a better prognosis than those with low PD-L1 expression. The median overall survival (mOS) was significantly longer [7]. However, it has also been reported that patients with negative PD-L1 expression can benefit from immune checkpoint inhibitors (ICIs) treatment, making PD-L1 a controversial biomarker [8]. Additionally, for patients with a tumor proportion score (TPS) ≥ 50%, pembrolizumab monotherapy is an alternative strategy.

The clinical significance of PD-L1 expression varies in NSCLC with different histological subtypes. In contrast to adenocarcinoma, PD-L1 expression has not been found to be significantly associated with immunotherapy efficacy in squamous cell carcinoma and small cell lung cancer (SCLC) to date [9]. In addition, Pisapia et al. found that approximately 55.7% of patients with positive PD-L1 expression (≥ 1%) had at least one genomic alteration [10], and the status of oncogenic driver mutations affected the patient's response to ICIs treatment [11].

## Detection of PD-L1 expression from different sources

### Detection of PD-L1 expression in tissues

#### IHC

IHC is the main method for detecting PD-L1 expression. Thin slices of lung cancer tissue stained with specific PD-L1 antibodies were used to visualize PD-L1 expression under a microscope. To date, the detection of PD-L1 expression using IHC relies on four primary antibodies: Dako PD-L1 IHC 22C3 pharmDx and Dako PD-L1 IHC 28–8 pharmDx from Agilent Technologies; the VennTANA PD-L1 (SP142) and VennTANA PD-L1 (SP263) assays from Roche.

PD-L1 IHC results are typically reported as the percentage of tumor cells or immune cells that express PD-L1 at a specific intensity, which in turn reflects the response to ICIs. 22C3 and 28–8 correspond to the Dako diagnostic platform. The positive standard of the former stipulates  ≥ 1% tumor cells. The staining of tumor cells was positive,  ≥ 50% of tumor cells were strongly positive, and  ≥ 1% of tumor cells were positive for the latter marker. SP142 and SP263 correspond to the Ventana diagnostic platform. The positive standard for the former is  ≥ 10% tumor and/or tumor-infiltrating immune cell staining, and that for the latter is staining  ≥ 25% tumor cells. Typically, at least 100 tumor cells are required to determine TPS.

Immunohistochemistry is widely used for PD-L1 detection because of its efficiency and stability. Notably, immunohistochemistry detection may yield false positives. This may be due to confusion between PD-L1-positive lymphocytes and histiocytes interspersed with PD-L1-negative tumor cells. In addition, granular cytoplasmic staining in nonmembranous malignancies may also be incorrectly interpreted as positive [12]. Currently, there is no gold standard for assessing the expression levels of PD-L1 in tumors and the TME. Further improvements in immunohistochemistry and the development of other PD-L1 detection technologies are still needed. In addition, since PD-L1 is partially expressed in tumor cells as well as in normal epithelial cells and lymphocytes, to improve the accuracy and resolution of staining results, multiplex immunohistochemical staining techniques can be utilized. These involve simultaneous labeling of antibodies against immune cells along with PD-L1 antibodies. This approach is highly important for assessing the status of the tumor immune environment and predicting the efficacy of immune therapy.

Immunofluorescence (IF) has been applied in the field of PD-L1 detection in recent years [13]. The principle of IF is similar to that of IHC. During this process, the antibodies are first labeled with fluorescein, and then the labeled antibody is bound to the corresponding antigen. Fluorescence microscopy was then used to determine the location of the antigen. Multiplex immunofluorescence (mIF) staining can quickly and reproducibly identify multiple molecular markers in the tumor microenvironment. Yaseen et al. confirmed that mIF has greater detection accuracy than does IHC or IF, which demonstrates its potential in clinical practice [14].

#### Glycosylation of PD-L1 and improved IHC

Many studies have demonstrated that the PD-L1 protein undergoes extensive N-glycosylation. Glycosylation, a PTM of membrane-bound proteins, regulates protein biosynthesis, folding, and stability by affecting protein structure and interactions with other molecules. It also plays a role in processes such as signal transduction, immunosuppression, and angiogenesis. However, the spatial hindrance of the large molecular structure of N-glycans may affect the recognition of PD-L1 by antibodies, leading to errors in the immunohistochemical detection of PD-L1 [15]. A study conducted by Heng Huan Lee et al. demonstrated that enzymatic removal of the glycan moiety on cell surface antigens significantly improved the affinity and signal intensity of anti-PD-L1 antibody binding. This deglycosylation process resulted in more accurate quantification of PD-L1 and improved clinical prognostic predictions [16]. Notably, this proposed sample deglycosylation method can be used to directly incorporate proteins into conventional immunohistochemical sample preparation via enzymatic reactions to eliminate protein n-junction glycosylation prior to antibody detection. However, the validation of this method needs to be accompanied by prospective data and standard operating procedures. Improving traditional IHC technology may be a new direction for improving the predictive efficacy of immunotherapy.

To overcome the masking effect of posttranslational modifications on PD-L1, fluorescence probes and targeted drug probes have also been extensively researched and applied for protein-specific detection [17, 18]. Compared to other targeted detection methods, such as gene sequencing and radioactive tracers, fluorescence probes offer unique advantages, including high selectivity, sensitivity, spatial and temporal resolution, low cost, and ease of operation.

### Detection of PD-L1 in peripheral blood

The utilization of circulating biomarkers in “liquid biopsy” has progressively garnered researchers' attention due to their non-invasive nature, dynamic monitoring capabilities, and ability to reflect the overall characteristics of the TME.

Soluble PD-L1 (sPD-L1) is the soluble form of the PD-L1 protein produced by tumor cells or other immune cells. It binds primarily to extracellular vesicles (exosomal PD-L1) or is expressed on the surface of circulating tumor cells (CTCs PD-L1). It can also exist in different forms, such as in its free form or bound to other proteins. Unlike PD-L1, which is expressed on the cell membrane, soluble PD-L1 does not require cell surface expression and can freely circulate in body fluids. sPD-L1 retains the programmed death protein 1 (PD-1)-binding domain and can competitively bind to PD-1 receptors, inhibiting the activation and function of T cells. It can, to some extent, represent the body's response to immune therapy. A study conducted by Zhou J et al. confirmed that the serum level of sPD-L1 in stage IV melanoma patients was elevated compared to that in healthy individuals and was associated with an increased likelihood of disease progression [19].

Notably, although many studies have indicated the clinical relevance of PD-L1 positive CTCs in various cancers, including NSCLC [14, 20–23], there is still controversy, and the analysis of CTCs is dependent on the use of different clones of anti-PD-L1 antibodies and CTCs enrichment techniques. Janning M et al. examined CTCs PD-L1 expression in 127 samples. They found that PD-L1 + CTCs increased in all patients as the disease progressed. However, the percentage of PD-L1 + CTCs was not related to the percentage of PD-L1 + tumor cells observed through IHC in the primary tumor tissue [24]. Furthermore, Kulasinghe and colleagues conducted a study utilizing blood samples from both head and neck cancer (HNC) patients and NSCLC patients for measurement of CTCs enrichment and IF staining of cell surface antigens. PD-L1-positive CTCs were found in 11 HNC patients (54.4%) and 11 NSCLC patients (64.7%). PD-L1 + CTCs were significantly associated with poor prognosis in HNC patients but not in advanced NSCLC patients [20].

Exosomes are small vesicles released by cells that contain various components, such as membrane lipids, proteins, and nucleic acids. They can be transferred to other cells through body fluids and facilitate intercellular communication. Research on the use of exosomes as novel diagnostic and prognostic biomarkers is pertinent for multiple diseases, including pancreatic cancer, ovarian cancer, breast cancer, and lung cancer [25–27]. Exosomal PD-L1 can mediate the therapeutic response of tumors to anti-PD-1/PD-L1 antibodies. It serves as a biomarker for monitoring disease progression and immune activity in NSCLC patients (Table 1). Compared to those in circulating tumor DNA (ctDNA) and CTCs, exosomes in peripheral blood have higher concentrations, good biological stability and compatibility, low immunogenicity, and low toxicity. Although there is no consensus on standardized exosome detection methods, various emerging detection methods have shown promising results [28, 29].

**Table 1:** Exosomal PD-L1 expression predicts the response to immunotherapy in NSCLC patients

Author year	Sample size	Line of ICI	Treatment	Exosome detection method	Endpoint	Conclusion
Yuting Wang [30]	149	1/2/more	Immunomonotherapy and ICI combination therapy	Enzyme-linked immunosorbent assay (ELISA)	Progression-free survival (PFS), disease control rate (DCR)	The expression level of exosomal PD-L1 was lower at pretreatment or the max fold increasing change was higher at 3–6 weeks; had a higher DCR and longer PFS
Shayista Akbar [31] 2023	17	–	Immunomonotherapy or chemotherapy-immunotherapy	–	Tumor response, best overall response (BOR)	Exosomal PD-L1 is potential biomarker to monitor response to ICIs therapy and can predict the clinical outcomes in NSCLC patients
Yoshihisa Shimada [32]	120	2	17 patients underwent postoperative recurrence and anti-PD-1 treatment	ELISA	recurrence-free survival (RFS), DCR	Exosomal PD-L1 levels were significantly correlated with tumor PD-L1 levels and the number of CD8 + tumor infiltrating lymphocyte (TIL), helping to predict anti-PD-1 response and clinical outcomes in patients with NSCLC
Yang Q [33]	21	–	Anti-PD-1/anti-PD-L1 therapy	Simoa™ PD-L1 Reagent Kit	PFS, Overall survival (OS), BOR	Increased expression of PD-L1 mRNA, exosomal PD-L1, or both in early stage of ICI treatment could serve as positive biomarkers of efficacy and OS in advanced NSCLC patients

#### ELISA

ELISA is a classic method for detecting proteins in body fluids. This method can be divided into indirect detection, double-antibody sandwich detection and competitive binding detection methods. ELISA has a simple and standardized operation process, good stability and reproducibility, and is the first choice for sPD-L1 detection [30, 34].

In recent years, with in-depth research on sPD-L1 and its innovation in antibody synthesis, great progress has been made in the use of the double-antibody sandwich ELISA method. A Japanese study developed a new ELISA test system that has higher detection sensitivity in NSCLC patients than conventional ELISA. The PD-1-Ig fusion protein replaced the immobilized capture antibody in conventional ELISA and was able to quantitatively detect functional sPD-L1 with the ability to bind to PD-1 at 75 °C [35]. To improve antibody immobilization, Sareh Zhand et al. introduced a protective coating that made the detection limit and sensitivity 225 times and 15.12 times greater than those of commercial ELISA kits, respectively [36].

#### Flow cytometry

Flow cytometry (FCM), which is used to detect single cells, can also be used to detect exosomes. Whiteside's team used antibody-carrying droplets to bind to PD-L1-containing exosomes and analyzed the number of PD-L1-positive exosomes by FCM. They confirmed that tumor progression is related to the expression level of exosomal PD-L1, which is similar to what has been observed via IF [27]. In a study on pancreatic cancer, Alexander lux et al. used immunocytochemistry and FCM to explore the feasibility of circulating exosomal c-Met and PD-L1 as markers for the diagnosis and poor prognosis of pancreatic cancer [37]. To date, several flow cytometry methods, such as the Apogee A50 microflow cytometer, which is more suitable for detecting exosome proteins than traditional analyzers, have been designed for exosome detection on the market [38].

#### Exploration of new detection methods

In addition to traditional detection methods, the development of new technologies has increased the prospects for the clinical application of sPD-L1. The predictive utility of PD‐L1 + CTCs in a chemotherapy setting has been investigated by Kallergi and colleagues. The authors used an isolation by size of tumor cells (ISET) technique developed by Rarecells Diagnostics SAS to isolate CTCs. Subsequently, Giemsa staining and immunostaining were used to detect PD-1 + /PD-L1 + CTCs in metastatic NSCLC patients before and after chemotherapy. The results revealed that PD-1 + CTCs, rather than PD-L1 + CTCs, had potential clinical significance [39].

In recent years, with in-depth research on exosomes and innovations in the synthesis of sPD-L1 antibodies, great progress has been made in identifying exosomal PD-L1. Compared with traditional IHC, innovative photochemical and electrochemical immunoassay detection methods provide PD-L1 adaptors with better performance; these adaptors often have unique three-dimensional structures, are easy to modify, and have good stability and sensitivity [40, 41]. Huang et al. developed a uniform, small-volume, efficient, and sensitive exosomal PD-L1 quantification method. This method combines a newly evolved aptamer that can effectively bind PD-L1 and is less hindered by glycosylation. Therefore, it has higher sensitivity, faster reaction time and easier operation than the commonly used ELISA method [42]. Wei J et al. attached gold nanospheres (GNSs) to the bottom of an eight-well chamber slide to form a detection substrate. Then, the Cy5-labeled CD63 aptamer (i.e., capture probe) was modified on the GNSs through Au–S bonds. After the exosome-containing sample was added, the FAM-labeled PD-L1 aptamer (i.e., immunoprobe) was added to identify PD-L1 on the exosomes. Through three-color fluorescence colocalization of Cy5, DiI and FAM, high-sensitivity and high-reliability detection of PD-L1-overexpressing exosomes was achieved [43].

Surface-enhanced Raman scattering (SERS) is a commonly used Raman spectroscopic analysis method for the determination of samples adsorbed on the surface of colloidal metal particles. It has been applied in the biomedical field. Pang Y et al. proposed a method to capture and analyze exosomal PD-L1 directly from serum. Fe3O4@TiO2 nanoparticles are used to enrich exosomes, and then, based on personalized SERS signal analysis, exosomal PD-L1 is quantified, and the relationship between exosomal PD-L1 expression and immunotherapy response can be determined [44]. In addition, automatic centrifugal microfluidic disk systems and nanoplasma sandwich immunoassays, have also begun to be used for the detection of exosomal proteins, greatly improving accuracy [45, 46].

However, liquid biopsy has many limitations. The origin of sPD-L1 has not yet been determined. In contrast to tissue PD-L1 analysis, most liquid biopsy analyses for sPD-L1 use different detection antibodies with variable identification capabilities [19, 47]. The value of sPD-L1 analysis in clinical practice largely depends on the sensitivity and error rates of the separation and detection techniques used [23]. However, sPD-L1 testing is indeed a clinical alternative, especially for patients for whom adequate tumor specimens cannot be obtained.

### Genetic detection of PD-L1

Plasma PD-L1 mRNA contains many genetic materials similar to those derived from tumor cells. Some of these substances are involved in cell cycle regulation, and some are related to important cellular activities, such as chromosome segregation, proliferation and migration. The prognostic prediction effect of extracellular vesicle (EV)-mRNA biomarkers may be better than that of EV-based protein biomarkers. Simonsen et al. evaluated PD-L1 expression in tumor samples from 42 NSCLC patients by IHC and detection of the PD-L1 encoding gene *CD274*. The results showed that gene-based assessment of PD-L1 expression was also consistent with the initial IHC assessment [48]. Another study used quantitative reverse transcription polymerase chain reaction (RT-qPCR) to detect the relationship between four target immune genes, *CD274*, programmed cell death 1 ligand 2 (*PDCD1LG2*), *CD8A* and interferon regulatory factor 1 (*IRF1*)*,* and ICIs treatment response in 122 patients with advanced NSCLC. Researchers have shown that high mRNA encoding immune checkpoint protein expression levels are associated with improved long-term survival and OS. Most importantly, low PD-L1 mRNA levels have a strong negative predictive value for the absence of long-term benefits [49]. One study proposed an immunogold biochip as a noninvasive alternative to quantify the expression of PD-L1 protein and mRNA on vesicles. The method is 1,000 times more sensitive than conventional ELISA and RT-qPCR [50].

PD-L1 mRNA, a gene-level biomarker, has great potential for guiding immunotherapy and predicting efficacy [51]. There are still differences between the transcription of the mRNA and the actual translation of the protein, which can lead to errors in test results. Whether this approach can compensate for limitations such as insufficient clinical specimen collection requires further experimental research and data analysis.

### Imaging detection of PD-L1

Standard methods for detecting PD-L1 on tissues, such as immunohistochemistry and ELISA, are often insufficient to accurately and comprehensively evaluate the expression of PD-L1, and the measurement of circulating markers lacks representativeness. Research on radiomics provides new ideas to solve this problem, achieving accurate, comprehensive and dynamic detection of PD-L1 expression levels in tumors through noninvasive methods. To date, nuclear medicine imaging relies mainly on positron emission tomography (PET) and single-photon emission computed tomography (SPECT). In terms of nuclear imaging detection of PD-L1, the development of new tracers is the key to this research. These tracers usually consist of two parts: radionuclides and PD-L1 ligands (monoclonal antibodies, monopolar antibodies, connexins, phages, peptides and small molecules).

#### Radiolabeled anti-PD-L1 antibodies

Radiolabeled anti-PD-L1 antibodies are currently the most commonly used type of nuclear medicine imaging probe. Anti-PD-L1 antibodies, such as thallium-99 m (99mTc) or technetium-99 (99Tc), are labeled with radioactive isotopes to observe their distribution in the body through PET or SPECT. Due to their high specificity, high affinity and availability, monoclonal antibodies have been shown to be highly reliable PD-L1 ligands. In 2015, Natarajan et al. developed a tracer based on the anti-mouse PD-1 monoclonal antibody 64Cu-DOTA-PD-1 and used PET imaging tracers to detect the expression of PD-1 in vivo for the first time [52]. Bensch et al. conducted the first human study to evaluate the feasibility of PET with zirconium-89-labeled atezolizumab in 2018. Preliminary results suggest that the assessment of PD-L1 status via molecularPET may better predict patient clinical response than can the use of predictive biomarkers from immunohistochemistry or RNA-seq [53].

#### Radiolabeled small molecule ligands for PD-L1

Compared with the shortcomings of antibodies, such as large molecular weight, long half-life, and slow generation of high-contrast images, some current nonmonoclonal antibody PD-L1 small molecule ligands, including connexins, appendages, peptides, small proteins, and antibody fragments, may act as tracers with lower molecular weights, higher tumor uptake rates, and rapid clearance.

Broos et al. developed the small molecular weight (15 kDa) single-domain antibody sdAb K2, which has high specificity for PD-L1. SPECT/CT images with a high signal-to-noise ratio could be generated within 1 h in mice. Notably, the sdAb K2 also has the ability to antagonize the interaction between PD-1 and PD-L1, which has potential therapeutic value [54]. In addition, the recently emerged immuno-SPECT probe [99mTc] Tc-HYNIC-KN035 also performed well in assessing the expression of PD-L1. The probe used was the nanobody KN035, which has high specificity and affinity for PD-L1, coupled to the chelator succinyl 6-hydrazinonicotinate and subsequently labeled with the radionuclide 99mTc [55]. Compared with other reported tracers, [99mTc] Tc-HYNIC-KN035 exhibited sustained high tumor uptake and a satisfactory target-to-background ratio [56]. Donnelly et al. used the Adnectin molecule (BMS-986192), which can detect the expression of PD-L1 in animal tumor models, to develop a tracer with high binding affinity for PD-L1, 18F-BMS-986192 [57]. Subsequent small-molecule tracers, such as 68 Ga-BMS-986192 and 68 Ga-NODA-BMS986192, have been optimized by 18F-BMS-986192, which has stronger automation and better stability [58, 59]. In addition, small molecule compounds such as radioactively labeled PD-L1-binding peptides and phages can also bind to PD-L1 and be enriched in tumor tissues, thereby facilitating PD-L1 imaging [60–63].

Radionuclides, such as 68 Ga, 18F, 99mTc, 64Cu, 89Zr, 124I, and 125I, have been used to label ligands for the production of various tracers. 18F is commonly used for PET, while 99mTc is more commonly used for SPECT. The characteristics of radioactive elements themselves also cause problems such as a half-life that is too long or too short, delayed clearance, long imaging time, and excessive radiation dose to healthy organs [64]. Therefore, choosing the appropriate radionuclide is crucial when manufacturing tracers.

### Artificial intelligence (AI) model detection of PD-L1

With the development of AI technology, the “Internet + ” model is being increasingly applied in clinical medical research. In 2023, two academic centers conducted a retrospective, multicenter study on 385 patients with advanced NSCLC who were suitable for treatment with ICIs. They evaluated combinations of machine learning algorithms with different feature selection methods and constructed imaging-based PD-L1 and PFS prediction models using radiological features extracted from preprocessed CT scans. Researchers found that logistic regression with ReliefF feature selection and support vector machine (SVM) with analysis of variance (ANOVA) with test feature selection were the best models for predicting PD-L1 expression and PFS [65]. Additionally, Shani Ben Dori et al. used machine learning models to explore the risk of inaccurate diagnosis of PD-L1 expression. The results show that the proposed model can be used to identify the risk of PD-L1 prediction errors, with the error rate increasing significantly when the PD-L1 expression ratio approaches the threshold [66].

AI technology has opened a new field for the development of noninvasive biomarkers. Previous studies have also shown that it is feasible to use AI deep learning to evaluate PD-L1 expression in NSCLC and predict the response to ICIs. Patients with shorter survival times are quickly and noninvasively identified by AI models, allowing clinicians to effectively select alternative optimal treatment strategies [67].

## Factors influencing PD-L1 detection

### Antibodies

Antibodies differ in their detection platforms, interpretation criteria, and corresponding positive thresholds, leading to heterogeneity in PD-L1 detection results [68–70]. The Thoracic Oncology Program at Yale University utilized E1L3N and SP142 antibodies to measure PD-L1 expression in 49 NSCLC whole-tissue sections and corresponding tissue microarrays. This study revealed heterogeneity within the tumors and significant interassay variability or inconsistency in PD-L1 detection [68]. This could be due to differences in antibody affinity, limited specificity, or distinct target epitopes. Efforts to determine the clinical value of these observations are underway.

In addition, different scoring systems and cutoff values for different ICIs have been established by various diagnostic companies and regulatory authorities. This poses a challenge for clinical pathologists. The study by Sakata et al. showed that when the cutoff values for PD-L1 positivity were different, the sensitivity and positive predictive value of endobronchial ultrasound-guided transbronchial needle aspiration (EBUS-TBNA) specimens were significantly different from those of surgical specimens, which may lead to misclassification of PD-L1 status in EBUS-TBNA specimens [71]. Lee H–H et al. reported that 7–16% of NSCLC patients who could benefit from immunotherapy were excluded due to false-negative PD-L1 detection by conventional IHC in the range of 0–49% [16]. According to the consensus on immunohistochemical tests of PD‐L1 in solid tumors (2021 version), IHC detection of PD-L1 expression as a predictive biomarker for anti-PD-1/PD-L1 treatment needs to be based on the drug-disease-diagnostic (3D) analysis principle [72]. Screening beneficiaries requires the selection of specific immunotherapy drugs and specific PD-L1 immunohistochemistry detection methods for specific disease types and the use of a supporting detection system.

### Sample types

Approximately 26 to 40% of NSCLC patients exhibit significantly different PD-L1 expression scores in different regions of the same tumor. This heterogeneity may be attributed to genetic variations within tumor cells, differences in cell types, and variations in the local immune environment [73, 74]. A study involving 160 patients demonstrated an approximately 48% difference in PD-L1 expression between surgical resection specimens and matched biopsy specimens. Compared to studies involving larger surgical resection specimens, studies involving biopsy specimens underestimated the observed PD-L1 status in the entire tissue sample, with the testing error decreasing as the sample size increased. This confirmed that the heterogeneity of PD-L1 protein expression may be associated with sample volume [75]. Therefore, PD-L1 is typically quantified in histological specimens from NSCLC patients who are scheduled for immunotherapy.

However, with advancements in detection techniques, recent studies have confirmed the feasibility and effectiveness of using cytological samples for PD-L1 quantification [76]. In a study by Skov et al., PD-L1 expression was strongly correlated with cytological blocks (CT fine-needle aspiration, fine-needle aspiration, EBUS, and pleural effusion) and histological specimens (lobectomy, wedge resection, core needle, and mucosa biopsy from the lung) obtained from the same site in lung tissue [77]. Heymann et al. examined the expression of PD-L1 in 214 specimens obtained from 188 patients and found no significant difference in the positivity rate between cytological and histological specimens [78]. In addition to differences in specimen volume, discrepancies in PD-L1 detection may also exist between primary tumors and metastatic lesions. Wang et al. reported minimal heterogeneity in PD-L1 detection between surgical samples from the same site and lymph node aspiration samples. The proportion of metastatic lymph nodes in the mediastinum with a PD-L1 TPS ≥ 50% was significantly greater than that of primary lung tumors [79]. Table 2 summarizes the heterogeneity of PD-L1 IHC detection results among different sample types of NSCLC.

**Table 2:** Heterogeneity of PD-L1 IHC detection results among different sample types in NSCLC

Author Year	Sample size	PD-L1 cutoff point	Method/antibody	Sample	Statistical difference of PD-L1 positive rate (P)	Concordance of PD-L1 expression
Smith [80]	120	< 1%, 1%–49%, ≥ 50%	22C3 pharmDx	EBUS-TBNA versus histology		78%
Wang [81]	1419	< 1%, 1%–49%, ≥ 50%	22C3 pharmDx	Cytology versus small biopsies versus surgical specimens	< 0.01	
TOROUS [82]	232	< 1%, 1%–49%, ≥ 50%	22C3 pharmDx	Cytology versus surgical specimens		No bias
Heymann [78]	188	< 1%, 1%–49%, ≥ 50%	22C3 pharmDx	Cytology versus small biopsies versus surgical specimens	0.083	91%
Skov [77]	86	< 1%, 1%–49%, ≥ 50%	28-8pharmDx; 22C3pharmDx	Cytology versus histology	no bias	85–95%
Sakakibara [76]	97	< 1%, 1%–49%, ≥ 50%	EPR1161(2)	EBUS-TBNA versus transbronchial biopsy (TBB) versus surgical	0.086	No bias
M Ilie [75]	160	0–3 (Score by expression)	SP142	Small biopsies versus surgical specimens		48%

In clinical practice, especially regarding advanced NSCLC patients and situations where obtaining histological specimens is challenging, cytological specimens obtained via EBUS-TBNA could serve as a novel option. However, there is still a lack of high-quality evidence and standardized operating procedures to support this approach. In addition, the detection results can also be influenced by chemical detection conditions, tissue slide preservation methods, and sample fixation time [83, 84].

## PD-L1 combined with other biomarkers

Despite the great success of clinical trials of multiple anti-PD-1/PD-L1 antibodies, the low therapeutic efficacy of these agents remains to be resolved. For most cancer patients, the PD-1/PD-L1 pathway is not the only rate-limiting factor in antitumor immunity. The application of biomarkers related to gene expression profiles and TME such as TIL, tumor mutational burden (TMB), mismatch repair deficient (dMMR), etc. in combination with or replacing PD-L1 expression to predict the efficacy of immunotherapy has been widely studied [85]. In particular, microsatellite instability (MSI)-high/ dMMR status have been approved by the Food and Drug Administration (FDA) as biomarkers for stratifying patients, and any unresectable or metastatic solid tumors with MSI-high/dMMR could be treated with pembrolizumab [86].

Although many biomarkers have consistent correlations with ICIs response, none are completely sensitive or specific. A study of NSCLC patients treated with ICIs found that when PD-L1 or TMB was used alone as a predictor, the clinical benefit rate was similar (35.3% vs 29.4%). However, this rate increased to 50.0% when PD-L1 expression and TMB were considered as combined predictors. The researchers proposed that TMB and PD-L1 expression are independent influencing factors for better clinical benefit, and combining TMB and PD-L1 into a multivariate prediction model will produce greater predictive power [87]. Chester Kao et al. evaluated the predictive value of TMB, PD-L1, and neutrophil–lymphocyte ratio (NLR) for the response to ICIs in 88 NSCLC patients [88]. TMB has certain predictive and prognostic capabilities for clinical outcomes after immunotherapy, and the combined use of the three biomarkers can improve the predictive value. According to PD-L1 expression and TIL status, the tumor microenvironment can be divided into four subtypes. Michele et al. suggested that it may be more reasonable to formulate personalized anti-tumor strategies based on this stratification. Subtypes with high PD-L1 expression and TIL are more suitable for ICI treatment. The other three subtypes may need combined treatment strategies to enhance their antitumor effects [89]. Given the complex interactions within the tumor immune microenvironment, a comprehensive assessment of various biomarkers may be necessary to improve the prediction of response to ICIs. However, there is no consensus on the optimal combination of biomarkers to predict the outcome of immunotherapy.

## Conclusion

In conclusion, significant progress has been made in the field of PD-L1 detection methods for NSCLC, which is beneficial to guide immunotherapy decision-making and the advancement of precision medicine. Tissue provides a snapshot of a tumor at a given time and location, while liquid biopsies can capture dynamic intra-patient genomic heterogeneity, establishing a tumor's molecular profile at the onset of treatment and throughout the disease course. Gene-based testing and radioactive tracers provide multidimensional PD-L1 expression information, offering new insights into PD-L1 expression. Machine learning methods utilize large-scale patient data and diverse features to predict PD-L1 expression, facilitating more accurate personalized treatment decisions. These methods provide valuable insights into PD-L1 expression in different sources within the body.

However, most existing detection methods are affected by factors such as sample type, detection platform, and antibodies, and have poor correlation with the efficacy of immunotherapy. Exploring new, more sensitive methods, or establishing a joint detection system for PD-L1 and other biomarkers, can better predict the efficacy of immunotherapy. However, the predictive performance of the selected combinations needs to be tested and compared in prospective studies. In order to ensure the consistency and accuracy of PD-L1 detection results, it is necessary to further understand the role of PD-L1 in immunotherapy response and promote the standardization and stability of the detection process.

## Data Availability

Not applicable.
